# Active learning for efficient analysis of high-throughput nanopore data

**DOI:** 10.1093/bioinformatics/btac764

**Published:** 2022-11-29

**Authors:** Xiaoyu Guan, Zhongnian Li, Yueying Zhou, Wei Shao, Daoqiang Zhang

**Affiliations:** College of Computer Science and Technology, Nanjing University of Aeronautics and Astronautics, MIIT Key Laboratory of Pattern Analysis and Machine Intelligence, Nanjing 211106, China; College of Computer Science and Technology, Nanjing University of Aeronautics and Astronautics, MIIT Key Laboratory of Pattern Analysis and Machine Intelligence, Nanjing 211106, China; School of Computer Science, China University of Mining Technology, Xuzhou 221116, China; College of Computer Science and Technology, Nanjing University of Aeronautics and Astronautics, MIIT Key Laboratory of Pattern Analysis and Machine Intelligence, Nanjing 211106, China; College of Computer Science and Technology, Nanjing University of Aeronautics and Astronautics, MIIT Key Laboratory of Pattern Analysis and Machine Intelligence, Nanjing 211106, China; College of Computer Science and Technology, Nanjing University of Aeronautics and Astronautics, MIIT Key Laboratory of Pattern Analysis and Machine Intelligence, Nanjing 211106, China

## Abstract

**Motivation:**

As the third-generation sequencing technology, nanopore sequencing has been used for high-throughput sequencing of DNA, RNA, and even proteins. Recently, many studies have begun to use machine learning technology to analyze the enormous data generated by nanopores. Unfortunately, the success of this technology is due to the extensive labeled data, which often suffer from enormous labor costs. Therefore, there is an urgent need for a novel technology that can not only rapidly analyze nanopore data with high-throughput, but also significantly reduce the cost of labeling. To achieve the above goals, we introduce active learning to alleviate the enormous labor costs by selecting the samples that need to be labeled. This work applies several advanced active learning technologies to the nanopore data, including the RNA classification dataset (RNA-CD) and the Oxford Nanopore Technologies barcode dataset (ONT-BD). Due to the complexity of the nanopore data (with noise sequence), the bias constraint is introduced to improve the sample selection strategy in active learning. Results: The experimental results show that for the same performance metric, 50% labeling amount can achieve the best baseline performance for ONT-BD, while only 15% labeling amount can achieve the best baseline performance for RNA-CD. Crucially, the experiments show that active learning technology can assist experts in labeling samples, and significantly reduce the labeling cost. Active learning can greatly reduce the dilemma of difficult labeling of high-capacity nanopore data. We hope active learning can be applied to other problems in nanopore sequence analysis.

**Availability and implementation:**

The main program is available at https://github.com/guanxiaoyu11/AL-for-nanopore.

**Supplementary information:**

Supplementary data are available at *Bioinformatics* online.

## 1 Introduction

Recently, nanopore sequencing technology has been recognized as the most advanced third-generation sequencing platform due to its long read duration of macromolecules and high resolution of single bases (Kasianowicz *et al.*, 1996; Ying *et al.*, 2014). The nanopore sequencing platform contains two liquid-filled reservoirs connected by a single nanopore (Henley *et al.*, 2016). Molecules that are to pass through the nanopore can generate characteristic blockade currents that reflect their physicochemical properties and structural information. The ionic current and residence time (signal bandwidth) of each amino acid base in the nanopore are the primary detection signatures used for data analysis (Aksimentiev *et al.*, 2004; Majd *et al.*, 2010; Steinbock *et al.*, 2014).

In general, nanopore sensors can be divided into two main categories: solid nanopores and biological nanopores. Solid nanopores consist of solid materials that can be mass produced by semiconductor fabrication (Feng *et al.*, 2015). They play a vital role in DNA sequencing and protein detection (Traversi *et al.*, 2013). Another representative nanopore is the biological nanopore consisting of a series of transmembrane protein channels. Translocation of these molecules allows single molecules to be detected and sequenced (Smith *et al.*, 2015; Zhang *et al.*, 2015, 2017). Currently, transmembrane protein channels are widely used as biosensors: *Mycobacterium smegmatis* porin A (MspA), which consists of rigid *β*-barrel structures. In a previous work (Wang *et al.*, 2021), the authors used the nanocavity of an MspA nanopore for RNA tertiary structure profiling at the single-molecule level. The MspA nanopore can directly distinguish many low molecular weight RNA structures such as miRNA, overhanged siRNA, blunt siRNA, tRNA or 5s rRNA. Oxford Nanopore Technologies (ONT) has researched and developed a series of portable, low-cost and automation-friendly nanopore sensors (Castro-Wallace *et al.*, 2017; Hoenen *et al.*, 2016; Johnson *et al.*, 2017; Laver *et al.*, 2015; Loose *et al.*, 2016). One of the ONT nanopore sensors is the MinION, a 4-inch-long USB-powered device that contains 512 sensor arrays. Each sensor is connected to four biological nanopores incorporated into an electrically resistant artificial membrane.

Indeed, nanopore sequencing technology has played a crucial role in sequencing the entire human genome. Unfortunately, due to the inevitable signal distortion of the sequences generated by nanopore sequencing technology, it is more difficult to analyze the sequence data only by manual processing. Fortunately, with the advances in computing power and machine learning [e.g. convolution neural network (CNN), support vector machine (SVM) or random forest (RF)], it has become possible to analyze the long-read nanopore sequences. Machine learning analysis of sequences can completely outperform traditional complex manual processing methods, which is why it is widely used in the field of nanopore sequence analysis. Machine learning algorithms used in the field of nanopore can be divided into (i) Traditional methods: Kolmogorov *et al.* (2017) used machine learning (RF and SVM) to process the ionic current signals obtained from solid-state nanopore sequencing of a polypeptide chain. They showed that the signals obtained with a sub-nanometer pore were sensitive enough to recognize protein sequences. Jia *et al.* (2019) trained an SVM classifier to detect DNA methylation events from ONT original data. Schreiber and Karplus (2015) proposed a hidden Markov model that can segment and integrate nanopore data. In addition, Liu *et al.* (2019) trained an SVM classifier to detect N_6_-methyladenosine (m6A) RNA changes in nanopores with high accuracy. (ii) Deep learning methods: Farshad and Rasaiah (2020) designed a two-layer neural network with the Levenberg–Marquardt (LM) transfer function to analyze the silicon nitride (Si_3_N_4_) nanopore data. Ni *et al.* (2019) used deep learning to detect DNA methylation state from nanopore sequencing. Liu *et al.* (2019) used the deep recurrent neural network to detect the DNA base modifications on Oxford Nanopore sequencing data.

These machine-learning algorithms have enabled an unprecedented breakthrough in applying nanopore sequencing to various biological tasks. The success of the above methods is often based on repeated iterative training with huge annotation datasets. However, data annotation often requires high sample labeling costs (i.e. experts with extensive expertise are required for manual labeling). In order to realize the exponential acceleration of labeling efficiency, we are the first ones to introduce active learning (AL) (Balcan *et al.*, 2009) technology into the field of nanopore sequence analysis. AL is a machine-learning method that selects and labels complex samples to obtain a highly accurate predictive model at a limited cost (Duplyakin *et al.*, 2016; Smith *et al.*, 2018). AL has been applied in many interdisciplinary fields, such as drug discovery, material design and other emerging disciplines (Gong *et al.*, 2021; Jablonka *et al.*, 2021; Kusne *et al.*, 2020; Lookman *et al.*, 2019; Ueno *et al.*, 2021; Xin *et al.*, 2021). Since it is an emerging discipline, there is basically no relevant research in the field of nanopores. Due to the complexity of the original sequence, the sample labeling requires more manpower. The sequence contains not only the effective molecular sequencing signals but also the noise signals. For example, in the previous work (Guan *et al.*, 2022; Wang *et al.*, 2021), the obtained sequence signal contained six RNA molecule sequencing signals and one noise signal. The experimental results show that the shapes of the sequencing signals of the three RNA molecules are similar and the noise signals have all kinds of strange shapes. Due to the complexity of the original sequence (signal aliasing and noise confusion), sample labeling in the nanopore region requires more labor cost. In procedure of labeling this dataset, we not only need to filter out the noise signals from all sequencing signals but also classify all RNA sequencing signals. Among them, three types of RNA signals with small differences that can be easily misclassified. In order to apply AL technology to nanopore datasets, we try to make the labeled samples more accurate. To minimize the occurrence of these samples being incorrectly labeled, we asked three labeling experts to label the entire dataset. Brainstorm the samples with different labels and combine the suggestions of the three labels to make the label results as accurate as possible. The datasets from ONT also face the same dilemma, such as the famous barcode dataset (Bell and Keyser, 2016; Misiunas *et al.*, 2018). Due to the poor signal-to-noise ratio, peak amplitude variation, velocity variation and peak overlap, it is difficult to identify them easily. Moreover, there are 58 178 samples in the whole dataset, making the labeling extremely difficult for the specialist. AL aims to select the most valuable samples from the unlabeled dataset and give them to the oracle (e.g. human annotator) for labeling to maintain the performance and reduce the labeling cost as much as possible. Therefore, AL may become an effective algorithm for various biological tasks in the nanopore domain in the future.

To overcome the dilemma of labeling nanopore dataset, we apply the AL-based strategy to verify their effectiveness in the nanopore field. We apply the AL-based techniques to the RNA molecule classification dataset (RNA-CD) from previous work (Guan *et al.*, 2022; Wang *et al.*, 2021) and the open resource ONT barcode dataset (ONT-BD) (Bell and Keyser, 2016; Misiunas *et al.*, 2018). The main contributions of our work are listed below:

- First, we analyze the feature distribution and the distribution of the original data sample of our RNA dataset. Based on the data distribution conditions, we select the best sampling strategy to evaluate the effectiveness of AL.
- We compare different AL algorithms in the classification performance. The experimental results confirm the effectiveness of AL algorithms and reduce the labeling cost.
- We introduce the bias constraint to improve the AL sample selection strategy as the complexity of the nanopore data (with noise sequence).
- We apply the AL strategy to other nanopore datasets, which confirms that the AL strategy can be used in more biological nanopore scenarios.
- We add the threshold evaluator to evaluate the model is not locally optimal when optimal performance is achieved.

## 2 Materials and methods

This section will briefly explain the problem of nanopore data analysis and several AL algorithms, concretely including five essential contents (i) explicitly explain the problem of RNA type prediction and the ONT barcode classification; (ii) briefly recommend several commonly used AL algorithms and emphatically dive into the details of the margin sampling AL algorithm; (iii) briefly expound on the AL strategy mechanism applied to the problem of RNA type prediction.

### 2.1 Nanopore problem statement

Before studying nanopore data, we need to depict the related concepts of nanopore data. Obviously, nanopore data are considered as a continuous time series, which means that several concepts of processing time series can be mapped to the nanopore data analysis. For the problem of RNA type prediction, the origin of the data is generated by sequencing six RNA molecules in the nanopore devices.

In general, supposing that the symbols *S* represent the original time series and *T* represent the RNA types. The input long sequence *S* is truncated to *n* sub-sequence $s=[s_{1},{\;s}_{2},\;\ldots ,{\;s}_{n}$], and the input RNA type *T* is truncated to *n* sub-targets $t=[t_{1},\;t_{2},\;\ldots ,\;t_{n}$]. It considers that the previous paper used the RF algorithm as the classification model to distinguish the RNA types (Guan *et al.*, 2022; Wang *et al.*, 2021). Therefore, the input of RF algorithm is the feature vector (***v***_*i*_) of the sub-sequence *s_i_* extracted by feature extract methods, which contains the length, mean, standard deviation and other statistical information. Traditionally, the RNA types prediction problem can be defined as a classification task:

(1) f:(vi,ti)→y,

where *y* is the predicted RNA type, and *f* is the mathematically rigorous function mapping (i.e. the RF algorithm for the problem of RNA types prediction).

In contrast, the data type of the ONT barcode classification problem is the same (Bell and Keyser, 2016; Misiunas *et al.*, 2018). However, the classification standard is different because it is not governed by the number of the current signal peaks in the ONT barcode data. For example, the first peak marks the beginning, three bits of uniquely identification molecules are designed, and the last peak indicates the end. The whole dataset has eight types of barcodes from ‘000’ to ‘111’. Misiunas *et al.* (2018) applied the CNN model to solve the specific problem, which can automatically extract features to distinguish the barcode types. Therefore, for an *L*-layer CNN model, the ONT barcode classification problem can be defined as:

(2) $$x_{l}=f^{\left(l\right)}(x_{l-1};\theta_{l}),l=1,\ldots ,L,$$

where *θ_l_* is the parameters of the *l*th layer and $f^{\left(l\right)}(x_{l-1};\theta_{l})$is the mathematically rigorous mapping function of the *l*th layer. The *x_l_* is the output of *l*th layer, and the *x_l-1_* is the input of (*l −* 1)th layer.

### 2.2 Active learning

#### 2.2.1 What is active learning

AL is a sort of machine learning whose overall process is: First, use the sample selection strategy to evaluate the ‘hard’ classification of sample data. Then, the selected samples are annotated manually by the field specialists with a high degree of professional knowledge. Finally, the model is retrained with the all-labeled samples to gradually improve the effectiveness of the model. Concretely, it can be summarized that AL is considered as an objective specification, which integrates human experience into the machine learning model. The AL strategy can be described as follows:

(3) A= (L, C, Q, U, S),

where *C* is the classifier to solve the specific task, *L* is the initial labeled sample pool for training the model *C*, and *U* is the unlabeled sample pool to query. *Q* is a query function, which is used to query a part of samples with a large amount of information within *U*. *S* is a supervisor, which can correctly label samples from *U*. The process can be summarized as follows: classifier *C* starts learning the initial labeled samples *L* through a small amount of information, selects one or a group of the most valuable samples from unlabeled sample pool *U* through a specific query function *Q*, asks the supervisor *S* for labels and then uses those newly labeled samples to train the classifier and execute the next round of query. The overall process is round until the pre-configuration stop condition is reached.

In the field of AL, the key is to select the appropriate annotation candidate dataset for manual annotation. This method is referred to as Query Strategy (QS). QS can be briefly summarized as the following six commonly used strategies: (i) Uncertainty Sampling; (ii) Query-by-committee-based queries; (iii) Query based on Expected Model Change; (iv) Expected Error Reduction based query; (v) Query based on Variance Reduction; (vi) Query based on Density Weighting Methods.

QS can be based on a single machine learning model or multiple machine learning models, depending on the actual situation. On the whole, the significance of the AL is to reduce the labeling cost and rapidly improve the effectiveness of the model. Therefore, the AL strategy can be simplified by selecting the best QS for the specific task.

In recent years, active learning has been widely studied using different approaches to address the data problem. Classical active learning approaches use either pool-based or query synthesis methods. In query synthesizing approaches, generative models are used to find the most informative samples (Mahapatra *et al.*, 2018; Mayer and Timofte, 2020). Pool-based methods also fall into several categories: uncertainty-based (Beluch *et al.*, 2018; Collins *et al.*, 2008; Joshi *et al.*, 2009; Wang *et al.*, 2017; Yoo and Kweon, 2019), representation-based (Sener and Savarese, 2017), and more recently a combination of the two (Sinha *et al.*, 2019; Zhang *et al.*, 2020). Pool-based active learning has been successfully used in many deep vision tasks. Meanwhile, theoretical dropout-based frameworks have also been used to measure uncertainty (Gal *et al.*, 2017).

#### 2.2.2 Uncertainty sampling

Uncertainty sampling is one sort of QS that selects the ‘indistinguishable’ sample data through the model and provides those select samples to the field specialist for annotation. The key to the uncertainty sampling method is how to describe the uncertainty of samples. Uncertainty sampling commonly contains three select ways: (i) Least Confident, (ii) Margin Sampling and (iii) Entropy Method.

The Least Confident method is to select those samples with the lowest probability and mark them with the mathematical formula:

$${\;x}_{\mathrm{LC}}^{*}={\mathrm{argmin}}_{x}\left(1-P_{\theta }\left(\left.\hat{y}\right|x\right)\right)={\mathrm{argmin}}_{x}\left(P_{\theta }\left(\left.\hat{y}\right|x\right)\right),\;(4)$$

where ${\hat{y}=\mathrm{argmin}}_{y}\left(P_{\theta }\left(\left.y\right|x\right)\right)$, *θ* represents a trained set of machine learning model parameters. For *x*, the $\hat{y}$ is the category with the highest probability of model prediction. The Least Confident method considers those sample data with the highest probability of model prediction but low confidence.

Margin sampling refers to selecting data samples that can easily be categorized into two categories or have a similar probability of being categorized into two categories. Edge sampling is to select the sample with the slightest probability difference between the largest and the second-largest predicted by the model, which is described by the mathematical formula:

(5) $${\;x}_{M}^{*}={\mathrm{argmin}}_{x}\left(P_{\theta }\left(\left.{\hat{y}}_{1}\right|x\right)-P_{\theta }\left(\left.{\hat{y}}_{2}\right|x\right)\right),\;$$

where$\;{\hat{y}}_{1}$ and ${\hat{y}}_{2}\;$represent the model predicts the largest possible class and the second-largest possible class for *x*, respectively.

In mathematics, entropy is used to measure the uncertainty of one system. A higher entropy value means the enormous uncertainty of the system, and contrary, lower entropy means less uncertainty. Therefore, for Entropy Method, sample data with high entropy can be selected as undetermined annotation data, which the mathematical formula can express:

(6) $${\;x}_{H}^{*}={\mathrm{argmin}}_{x}(\sum_{i}P_{\theta }(\left.y_{i}\right|x)\cdot \ln P_{\theta }(\left.y_{i}\right|x)).\;$$

Compared with Least Confident and Margin Sampling, the Entropy Method considers the results of all categories of *x* determined by the model. While Least Confident only considers the maximum probability, the margin sample considers the maximum and the second-largest probabilities.

### 2.3 AL strategy in nanopore data

**Fig. 1.**
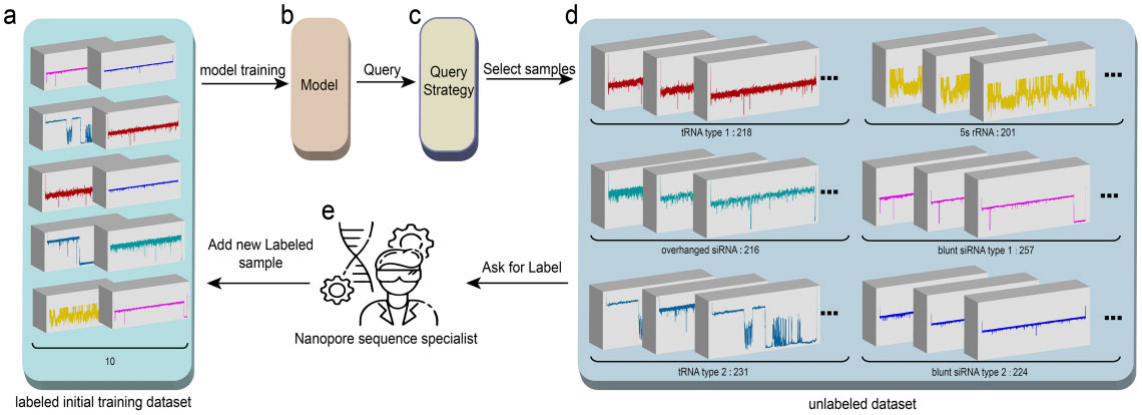
Schematic of AL strategy applied in RNA-CD. (**a**) The labeled samples pool *L*. (**b**) The machine learning model *C*. (**c**) The query function Q. (**d**) the unlabeled dataset pool *U*. (**e**) The nanopore field specialist *S* who can annotate the query sample. The numbers below each rectangular block in the figure indicate the total number of samples in each category

To further illustrate the application of the AL strategy in the nanopore field, we briefly describe the overall process of applying the AL strategy to RNA-CD, as shown in Figure 1. Without losing generality, the process of applying the AL strategy in ONT-BD is similar.

Concretely, the overall process of the AL strategy applied to RNA-CD consists of five parts (*L*, *C*, *Q*, *U*, *S*) in Eq. (3), corresponding to Figure 1a–e, respectively. In our experiment, the initial labeled sample pool *L* is set to ten samples to train the model, as shown in Figure 1a. In some cases, the machine learning model *C* is specially set as the RF algorithm in the RNA types prediction experiment, as shown in Figure 1b. Correspondingly, we set the C as the CNN model in the ONT barcode classification experiment and the RNA type classification experiment by S2Snet (Guan *et al.*, 2022). Notably, the query function *Q* contains six common strategies: query-by-committee (QBC) is based on the QS (Freund *et al.*, 1997), Random is the random sampling, QUerying Informative and Representative Examples (QUIRE) is the pool-based active learning strategy (Huang *et al.*, 2010), Density is the density-based sampling AL strategy (Nguyen and Smeulders, 2004), EER is Expected Error Reduction (Roy and McCallum, 2001), LAL is Learning Active Learning (Konyushkova *et al.*, 2017), SPAL is Self-Paced Active Learning (Tang and Huang, 2019) and UNCertainty sampling (UNC) is based on the Margin Sampling (Lewis and Gale, 1994) in our experimental configuration, as shown in Figure 1c. Obviously, the unlabeled dataset pool *U* is the rest of the training dataset without the initial 10 labeled samples, as shown in Figure 1d. The numbers below each rectangular block in the figure indicate the total number of samples in each category. Especially in the nanopore field, supervisor *S* is the nanopore field specialist with a high degree of professional knowledge of annotating the unlabeled sample, as shown in Figure 1c. The overall learning process is a continuous and iterative, which will stop when the best test performance (accuracy) is achieved.

### 2.4 Bias constraint for nanopore data

The peculiarity of nanopore data is that there may be some samples between categories that are difficult to accurately label or learn (noise sequence), and these samples sent to the model will affect the final classification task. Therefore, in the nanopore field, AL selects samples from the sample pool that are easy to label or learn. Here, we design a specific bias constraint for AL of nanopore data based on the idea in this article (Farquhar *et al.*, 2021).

Generally, the aim of the machine learning model is to find a decision rule $f_{\theta }$ corresponding to inputs, x, and outputs, y, drawn from a data distribution $p_{\mathrm{data}}(x,\;y)$ which, given a loss function $L(y,f_{\theta }(x))$, minimizes the risk:

(7) $$r=E_{x,\;y∼p_{\mathrm{data}}}\left[L\left(y,f_{\theta }\left(x\right)\right)\right].$$

In AL, we begin with a large unlabeled dataset, known as the pool dataset $D_{\mathrm{pool}}=\left\{x_{n}|1\leq n\leq N\right\}$, and sequentially pick the most useful M points for which to acquire labels. The empirical risk evaluated using the M actively sampled labeled points is:

(8) $$\tilde{R}=\frac{1}{M}\sum_{m=1}^{M}L\left(y_{m},f_{\theta }\left(x_{m}\right)\right).$$

Almost all studies of AL use this estimator, which is a biased estimator when the M points are actively sampled. Such bias is unavoidable at AL and is particularly evident in the nanopore field. There are two main reasons for this bias (i) Some samples are easily mislabeled (noise sequence); (ii) Some samples perform negative optimization of the model. Therefore, in nanopore field, the sampled M points should be easy for the model to learn or easy for the expert to label. First, we use the traditional t-SNE method for feature mapping, select the mean and variance of the mapped features, and set the center point of the existing training dataset for feature mapping as *O*. Then, we calculate the feature mapping distance between the center point *O* and each sample point in the sample pool $D_{\mathrm{pool}}$, which is defined as $W=\left\{w_{n})|1\leq n\leq N\right\}$. To adjust the weight balance, W must be normalized and reversed.

(9) $$W=1-\mathrm{normalization}\left(W\right).$$

The bias constraint is added to the unlabeled sample pool $D_{\mathrm{pool}}$ to form a new unlabeled sample pool $D_{\mathrm{pool}}^{'}=\left\{{(x}_{n},w_{n})|1\leq n\leq N\right\}$. The data points closer to the center of the feature points of the existing training dataset are given the maximum weight, and the data points farther from the center of the feature points of the existing training dataset are given the minimum weight (e.g. noise sequence, which can be easily mislabeled). The pseudocode in the Supplementary material is provided for better understanding. Before selecting samples in each round of AL, we need to reassign the weight to the sample pool $D_{\mathrm{pool}}$. In the nanopore field, we define this weight as a bias constraint that can avoid the extreme situation in the process of AL sample selection. This can further improve the performance of the test dataset for the data in the nanopore field.

## 3 Experiments

In the experimental section, first, we depict the two datasets (RNA-CD and ONT-BD) in Section 3.1 and briefly illustrate the experimental settings related to the dataset allocation and model configuration in Section 3.2. Secondly, we show the experimental results of six AL comparison methods in Section 3.3 and briefly analyze the strengths and weaknesses of the AL experimental results in the nanopore field in Section 3.4. Finally, in Section 3.5, we elaborate show the effectiveness of the AL in terms of loss of labeling time and difficulty of labeling.

### 3.1 Dataset

Before the AL experiment, we need to briefly present the dataset used to evaluate the effectiveness of AL. We primarily use two nanopore sequencing datasets in this work, RNA-CD and ONT-BD.

For RNA-CD, we use the RNA sequencing data from the previous publication (Guan *et al.*, 2022; Wang *et al.*, 2021). The data type is generally the time series shown in Figure 1. The sequenced analytes include four RNA types: tRNA, overhanged siRNA, 5S rRNA and blunt siRNA. Due to the RNA tertiary folding structure of the translocation pore, tRNA and blunt siRNA have two sequencing signals. Therefore, for RNA-CD, the number of categories is set to seven, including the six RNA sequencing signal categories for training the model and the one noise category to improve the robustness of the model. The data distribution is shown in Supplementary material SA.

For ONT-BD, the barcode data come from the paper (Bell and Keyser, 2016; Misiunas *et al.*, 2018). Unusually, the judgment basis for determining the category is determined by the number of current signal peaks from the ONT-BD. The number of categories includes eight barcode categories, from ‘000’ to ‘111’. Misiunas *et al.* (2018) used the CNN model to classify ONT-BD. To solve the problem of the unequal length of input sequences, uniform 700-point vectors are used as input, and the shorter sequences were padded with Gaussian noise at the end (*μ* = 0, *σ* = 0.072).

### 3.2 Experimental settings

As mentioned above, two representative datasets are selected for our experiments, namely RNA-CD and ONT-BD. The dataset RNA-CD includes 1020 training samples and 559 test samples (Wang *et al.*, 2021). In the previous paper (Guan *et al.*, 2022), we extended the dataset to 1388 training samples and 1387 test samples for the Deep Learning requirements. Accordingly, the ONT-BD includes 52525 training samples and 3464 test samples.

In general, the goal of the AL strategy is to reduce the sample number of the training dataset while maintaining the best monitoring performance. The best performance in classifying RNA type is 0.934, as reported in the previous work (Wang *et al.*, 2021) using the classical machine learning algorithm RF. In the previous work (Guan *et al.*, 2022), the best performance in classifying RNA type was increased to 0.957 by using the deep learning algorithm S2Snet. Therefore, in this work, the RF and S2Snet models are used to verify the active learning strategy, and the parameter configuration and running environment are the same as the previous work (Guan *et al.*, 2022; Wang *et al.*, 2021).

The best performance of the ONT barcode classification task is 0.946, as reported in the paper (Misiunas *et al.*, 2018). They proposed the deep learning model QuipuNet extract features and classify barcodes automatically. Because QuipuNet is a model based on a convolutional-neural-network, we use similar training parameters and experimental settings in this article. The AL code is developed based on the AL framework (Tang *et al.*, 2019). The model is trained on a GPU (Nvidia GeForce GTX 2080 TI). To minimize the loss function, the same Adam optimization algorithm is used (LR = 0.001; decay =0.97; the batch size of 64).

QBC, QUIRE, Density, LAL, SPAL, EER and UNC are the baseline method be used to evaluate the effectiveness of AL. To avoid the influence of model parameters on the experimental results, we use default parameters for each baseline model. For the QBC, the parameter setting select the ‘query by bagging’ and the disagreement select ‘vote entropy’, the setting is the same as the paper (Freund *et al.*, 1997). For QUIRE, the parameter setting is *λ* = 1, the kernel is ‘rbf’ and *γ* = 1, the setting is the same as the paper (Huang *et al.*, 2010) because these parameters have the best performance. For the Density, the parameter setting is ‘manhattan’ metric and the kernel is ‘gaussian’, the setting is the same as the paper (Nguyen and Smeulders, 2004). For the LAL, the parameter setting is ‘cls_est’ = 50 (The number of estimator used for training the random forest) and ‘train_slt’ = True (Whether to train a selector in initializing), the setting is the same as the paper (Konyushkova *et al.*, 2017). For the SPAL, the parameter setting is *γ* = 1, the initial value of *λ* = 0.1 and the kernel is ‘rbf’ whose kernel coefficient is 1, the setting is the same as the paper (Tang and Huang, 2019). For the UNC, the implementation of the uncertainty measure is entropy, since it takes into account the results of all categories determined by the model.

In this article, we primarily use two critical metrics to evaluate the effectiveness of the different AL strategies on the nanopore data. The SavedRate (SR) is the first one, indicating that the labeling cost of the AL method is reduced compared to the full complete sample (FS). The SR can be defined as follow:

(10) $$\mathrm{SR}=1-\frac{\mathrm{EA}}{\mathrm{FS}},$$

where ExpertAnnotated (EA) represents the number of labeled samples by specialists when the model reaches the given target performance, FS represents the number of unlabeled samples provided by the current dataset, and the number of labeled samples used when training with full samples. Another metric is Time, which represents the time required to achieve the best performance.

### 3.3 Experimental results

We report experimental results from two datasets (three models) on the different AL sampling strategies. The eight AL sampling strategies: QBC, Random, QUIRE, Density, LAL, SPAL, EER and UNC. We perform a series of experiments to show the performance of the two datasets (three models) with the different AL sampling strategies. The results of the Confusion Matrix are shown in Supplementary Material SE.

**Fig. 2.**
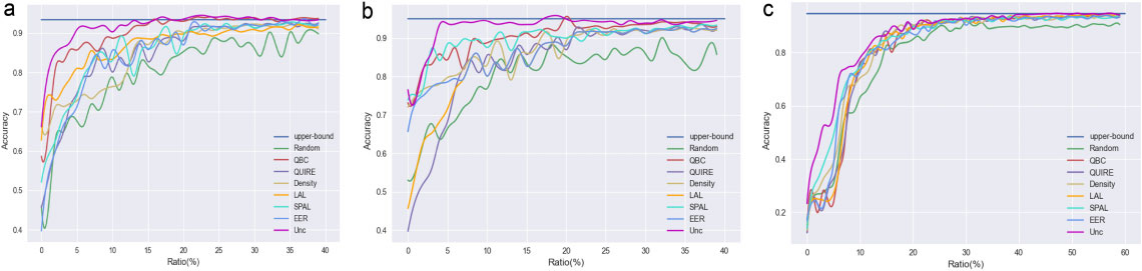
The experimental results of the model trained with the selected sample by AL. (**a**) Test set AUC of final classifiers versus number of iterations on RNA-CD (RF). (**b**) Test set AUC of final classifiers versus number of iterations on RNA-CD (S2Snet). (**c**) Test set AUC of final classifiers versus number of iterations on ONT-BD

For the RNA type classification task, we first use the randomly selected initial 10 samples to train the RF and S2Snet model, and use different sampling strategies to select ten samples for specific queries in each iteration. Therefore, the classification performance gradually improves as the iteration time increases. We first check the performance of the different AL sampling strategies for two nanopore data. The results show that UNC performs better than other methods, as shown in Table 1. The first metric is SR, which represents the percentage of labeling cost reduction of the AL method compared to the full sample. The second metric is the time, i.e. the runtime required to achieve the best performance. The relationship between the accuracy of the test set of the final classifier and the number of iterations is shown in Figure 2a and c. By measuring the accuracy of the test set of the final classifier model, the performance of UNC on RNA-CD is significantly better than that of other methods and is in line with the best performance. UNC quickly achieves the performance of the final model trained on the full number of ground truth training labels when the number of labels is about 15%.

**Table 1.** The performance comparison between the AL strategy at RNA-CD and ONT-BD

Methods	RNA-CD (RF)		RNA-CD (S2Snet)		ONT-BD (QuipuNet)	
	SR↑	Time↓	SR*↑*	Time↓	SR*↑*	Time↓
Random	0.012 ± 0.003	79.04s ± 2.3s	0.024 ± 0.001	32min42s ± 24s	0.058 ± 0.007	2h39min36s ± 35s
QBC	0.834 ± 0.021	762.58s ± 7.5s	0.836 ± 0.143	4h21min55s ± 10s	0.448 ± 0.063	17h39min18s ± 73s
QUIRE	0.023 ± 0.002	1288.06s ± 10.3s	0.031 ± 0.011	8h16min17s ± 36s	0.089 ± 0.001	26h56min34s ± 65s
Density	0.033 ± 0.005	101.58s ± 5.6s	0.037 ± 0.002	59m11s ± 8s	0.134 ± 0.021	3h41m23s ± 35s
LAL	0.045 ± 0.017	154.83s ± 3.4s	0.054 ± 0.017	1h15m21s ± 7s	0.198 ± 0.011	4h56m43s ± 42s
SPAL	0.563 ± 0.113	564.55s ± 6.3s	0.574 ± 0.158	3h43m25s ± 15s	0.334 ± 0.018	14h32m12s ± 51s
EER	0.322 ± 0.134	379.54s ± 8.2s	0.342 ± 0.129	2h11m43s ± 6s	0.212 ± 0.042	8h14m32s ± 32s
**UNC**	**0.844 **±** 0.045**	88.88s ± 1.4s	**0.855 ± 0.062**	40m10s ± 34s	**0.553 ± 0.033**	2h49m10s ± 21s

*Note*: For RNA-CD, the results of two methods (RF and S2Snet) are reported, respectively. Mean and standard deviation of performance on each benchmark are reported. The Bold indicates the performance is better than other method.

Figure 2c shows the number of iterations on the test sets AUC and ONT-BD of the terminal classifier. The results show that UNC significantly outperforms the other methods even on this dataset. UNC quickly approaches the performance of a terminal model trained on the full number of ground truth training labels when the number of labels is about 50%.

For RNA-CD datasets, only about 15% of the labeling rate is required to achieve the optimal performance. However, for ONT-BD datasets, 50% of the labeling rate is required to achieve the optimal performance. We analyze that there are two main situations that cause this difference: (i) The different capacity of the dataset. (ii) The task of the dataset is different. The task of ONT-BD is relatively difficult; the task of RNA-CD dataset is relatively easy.

From the experimental results, the performance curve becomes stable after reaching the optimal performance (upper-bound line), which also confirms that the results of the active learning algorithm in the field of nanopore are similar to those of other active learning applications. As the number of iterations increases, the performance does not vary much. For non-convex models, the local optimal performance is similar to the global optimal performance, and the global optimal cost is higher, so many existing models have not been optimized to the global optimal solution. For convex models, active learning (semi-supervised learning method) will not fall into local optimization. Therefore, when the performance of the model is not improving, it is a local optimal solution for the non-convex model and a global optimal solution for the convex model.

Obviously, the performance of UNC is better than that of other AL sampling methods. Moreover, the experimental results show that the AL strategies can effectively reduce the labeling rate of the samples and maintain the training performance for the full ground truth. Indeed, the AL strategies can be applied in the field of nanopores to reduce the cost of specialists.

### 3.4 Analysis

The use of active learning in nanopores has two goals. The first is to reduce sample labeling costs incurred by field professionals in manually distinguishing event categories. The second objective is to use AL to deepen the understanding of the selected samples to achieve the performance of the training model with complete ground truth. The experimental results show that the strategies of AL can accomplish the first goal. In this section, we primarily analyze the second objective: how to achieve the best performance of the subsamples selected by AL.

#### 3.4.1 Feature importance

**Fig. 3.**
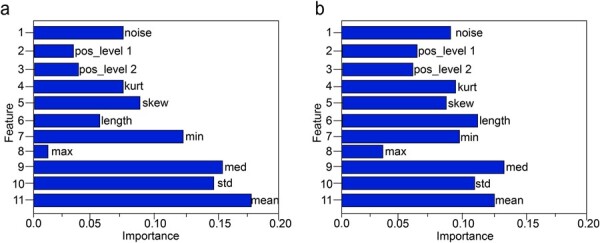
The feature importance of the results output. (**a**) The feature importance of full ground truth training model. (**b**) The feature importance of sample training model selected by UNC to achieve the performance of full ground truth training

In addition, it is interesting to analyze the feature importance of the results output by the full ground truth training model and the sample training model selected by UNC to achieve the full ground truth training performance, as shown in Figure 3a and b. The feature importance is generated during the model test, proving the relative importance of all 11 features in event detection. The horizontal axis shows the important indicators and the vertical axis shows the eleven features. According to the feature distribution analysis in Section 3.2, the feature ‘noise’, feature ‘standard deviation’ and feature ‘length’ change significantly. The UNC strategy reduces the difference in importance between the features.

#### 3.4.2 Example sequences

**Fig. 4.**
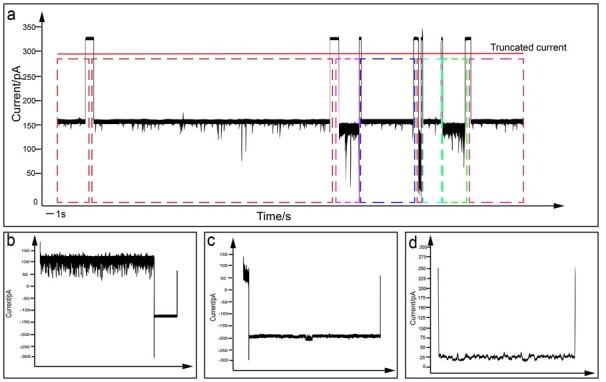
The schematic diagram of the sequence of RNA-CD. (**a**) Schematic diagram of RNA-CD long sequence segmentation. (**b–d**) Three representative noise signals after segmentation selected by the active learning strategy of UNC

The main function of the AL strategy is to select the whole sample set selectively. The above experimental results show that the AL method UNC achieves the best experimental performance. Therefore, we analyze the samples selected by the AL strategy UNC. In RNA-CD, the samples are obtained by truncating the long sequencing signal. However, due to the influence of the sequencing noise, there are many noise samples in the truncated signal, which are not conducive to the final classification performance. Eliminating the proportion of noise samples after truncation will play a very positive role in the final classification performance. Therefore, we investigate whether AL can guide the initial sequencing signal truncation. We select the samples selected by UNC active learning for analysis, of which Figure 4b–d shows one of the three most representative signal waveforms. It can be seen that these three signals are noise samples. For Figure 4b, most of the signals are concentrated near the blocking current of 0.5, which is easily misclassified as tRNA type 1.

#### 3.4.3 Sample distribution

Similarly, for Figure 4c, most of the signals are concentrated near the blocking current of 0.6, which can be easily misclassified as tRNA type 2. Figure 4d is a standard noise sample where most signals are concentrated near the blocking current of 0.1. From these samples selected, we can see that active learning selects more samples that the classifier can easily misclassify. This kind of sample signal gives us inspiration for truncating signals, i.e. when truncating signals, signals with extremely inappropriate sample signal span are actively sifted through the minimum boundary so as not to affect the final classification performance.

**Fig. 5.**
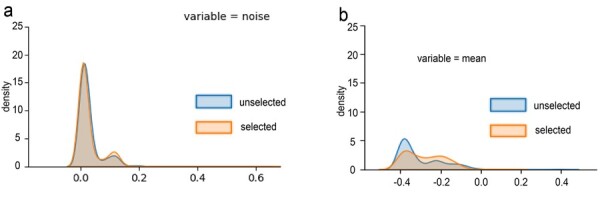
The schematic diagram of the sample distribution. (**a**) The RNA-CD sample distribution of the feature value, where the variable is the noise. The blue area is the unselected sample, and the orange area is the selected sample. (**b**) The ONT-BD sample distribution of the sample value, where the variable is the sample mean. The blue area is the unselected sample, and the orange area is the selected sample

When classifying RNA types, we perform the statistical analysis of the selected samples. As shown in Figure 5a, the distribution of the selected samples is similar to that of the unselected samples. In addition, the selected samples for the ONT barcode classification task have a more concentrated distribution, as shown in Figure 5b. The experimental results show that the AL sample selection strategy can select the samples with the best distribution that matches the distribution of the test dataset. The guideline that the AL strategies select the samples to be labeled can help biologists manually sort out the useful samples from the dataset for model training and give biologists better interpretability, which is the task of machine learning. More results are shown in Supplementary material SD.

### 3.5 Scalability

To verify the scalability, we use the other two nanopore datasets to implement the AL strategy and analyze how sample labeling costs can be reduced under the premise of ensuring optimal performance. The first dataset is from (Smith *et al.*, 2019), the author proposed a novel strategy for barcoding and demultiplexing direct RNA sequencing nanopore data that does not rely on basecalling or additional library preparation steps. The method is called DeePlexiCon and implements a 20-layer residual neural network that can demultiplex 93% of reads with 95.1% specificity. The dataset contains 160K samples for training, 40K samples for testing, and 40K samples for validation. Another dataset we use to check scalability from the paper (Wang *et al.*, 2019). O^6^-carboxymethylguanine (O^6^-CMG) is a highly mutagenic alkylation product of DNA that induces transition mutations relevant to gastrointestinal cancer. First, we cut the original data of the nanopores used in the paper (Wang *et al.*, 2019) and obtained a dataset of 1010 samples in total. Using the SVM classifier, the classification accuracy reaches 98%. To evaluate the scalability of AL on more nanopore datasets, we test the use of AL with two datasets for different tasks. For these two datasets, all AL baselines are configured as in Section 3.2. We perform the experiments with different AL baselines to select the samples that we literately train until the model test performs the best performance.

The experimental results are shown in Table 2. From the table, it can be seen that for the DeePlexiCon dataset, 52% of the AL labeling samples are required to achieve the optimal performance, while for the O^6^-CMG dataset, 22% of the AL labeling samples are required to achieve the optimal performance. This experimental result also guarantees that the AL strategy is universal and has good scalability for the universal nanopore dataset.

**Table 2.** The performance comparison between the AL strategy at DeePlexiCon and O^6^-CMG

Methods	DeePlexiCon		O^6^-CMG(SVM)	
	*SR↑*	Time↓	*SR↑*	Time↓
Random	0.047 ± 0.012	1h23min45s ± 21s	0.023 ± 0.006	60.83 s ± 1.5s
QBC	0.323 ± 0.043	6h25min46s ± 33s	0.776 ± 0.011	681.24s ± 4.3s
QUIRE	0.063 ± 0.007	4h31min51s ± 14s	0.036 ± 0.008	975.36s ± 7.4s
Density	0.098 ± 0.012	2h15m16s ± 54s	0.054 ± 0.005	87.26s ± 2.6s
LAL	0.145 ± 0.021	2h56m26s ± 12s	0.071 ± 0.018	136.37s ± 4.7s
SPAL	0.264 ± 0.013	5h13m22s ± 14s	0.432 ± 0.109	425.63s ± 8.1s
EER	0.156 ± 0.034	3h43m21s ± 12s	0.265 ± 0.084	253.13s ± 6.4s
**UNC**	**0.478 ± 0.013**	1h30m24s ± 11s	**0.782 **±** 0.035**	62.54s ± 2.3s

*Note*: The mean and standard deviation of performance at each benchmark are given.  The Bold indicates the performance is better than other method.

### 3.6 Ablation study

In this article, we use bias constraint to restrain the samples that are difficult to label or learn. We perform an ablation study on two variants of UNC, which is the best AL strategy for the three nanopore datasets. The metric is SR, which is the percent reduction in labeling cost of the AL method compared to the full sample. The results are shown in Table 3, from which we can draw the following conclusions: (i) bias constraint plays a significant role in improving the classification performance of RNA type; (ii) bias constraint also plays a significant role in improving the classification performance of ONT barcode; (iii) the result of using bias constraint is better than the result of not using bias constraint, which shows that bias constraint plays a significant role in improving the classification result.

**Table 3.** Results of the ablation study

Bias constraint	RNA-CD (RF)	RNA-CD (S2Snet)	ONT-BD
–	0.835 ± 0.031	0.847 ± 0.025	0.544 ± 0.021
√	**0.844 ± 0.045**	**0.855 ± 0.062**	**0.553 ± 0.033**

*Note*: The ‘√’ expresses that the bias constraint is used, whereas the ‘–’ expresses that the bias constraint is not used. For RNA-CD, the results of two methods (RF and S2Snet) are given, respectively. The AL strategy is UNC. The Mean and standard deviation of performance on each benchmark are reported. The Bold indicates the performance is better than other method.

### 3.7 Local optimal evaluation

In fact, because it is not possible to label all samples in the real world, so we do not know where the best performance of the model. When AL is in constant iteration, in order to stop the iteration, the basis of judgment is that the performance of the model is no longer improved. To ensure that the current model is not locally optimal, we set a threshold evaluator to evaluate the difference between the new sample selected in the current round of iteration and the new training dataset. If the difference is smaller than threshold value, the current model does not need to be trained further, and the model has reached the optimal performance. To achieve the above objectives, we carry out the following series of operations. First, we implement AL iteration to make the model reach the performance is no longer improved. Then, mapping the samples in the current training dataset to t-SNE according to the mean value and variance of the feature, construct a circle with center *O* and radius *r*, and mapping the 10 samples selected by the current AL to t-SNE according to the mean value and variance of the feature. Finally, the threshold *T* is evaluated according to the number of samples with the distance *d *>* r* between the mapping point and the center of the feature *O*. If the number of *d *>* r* is larger than *T*, there is a difference between the selected samples and the training dataset. The model needs to be trained for the new samples, which is not optimal for the model. If the number of *d* > *r* is less than *T*, there is no significant difference between the selected samples and the training dataset, and the model no longer needs to be trained. At this time, it can be ensured that the model is in the optimal situation, and the subsequent iteration will have little impact on the model. The number of categories *n* in each dataset is different, the threshold *T* needs to be adjusted according to the number of categories *n*. Through the verification of the experimental results of the three datasets, when the model performance is not improving, the threshold value is set to *T = n*/2, and the number of AL iterations obtained is similar to the number of AL iterations for the optimal performance obtained from the model training under full ground truth.

The experimental results are shown in Table 4. The model selected in the experiment is UNC, in which the first line is the SR when the AL reaches the optimal performance using threshold evaluation without knowing the full ground truth dataset. The second line is the SR when the AL iteration reaches the optimal performance when the full ground truth optimal performance is known. From the experimental results, the difference between SR is not significant when the full ground truth is known or not known, which also confirms the effectiveness of our selected threshold evaluator and the feasibility of the local optimal evaluation.

**Table 4.** Results of the local optimal evaluation study

Model	RNA-CD (RF)	RNA-CD (S2Snet)	ONT-BD
UNC(threshold)	0.843 ± 0.021	0.852 ± 0.016	0.552 ± 0.018
UNC	**0.844 ± 0.045**	**0.855 ± 0.062**	**0.553 ± 0.033**

*Note*: The ‘UNC(threshold)’ expresses that the threshold is used, whereas the ‘UNC’ expresses that the full ground truth is used. For RNA-CD, the results of two methods (RF and S2Snet) are given respectively. The AL strategy is UNC. The mean and standard deviation of performance on each benchmark are reported. The Bold indicates the performance is better than other method.

### 3.8 Experiment for labeling cost

**Fig. 6.**
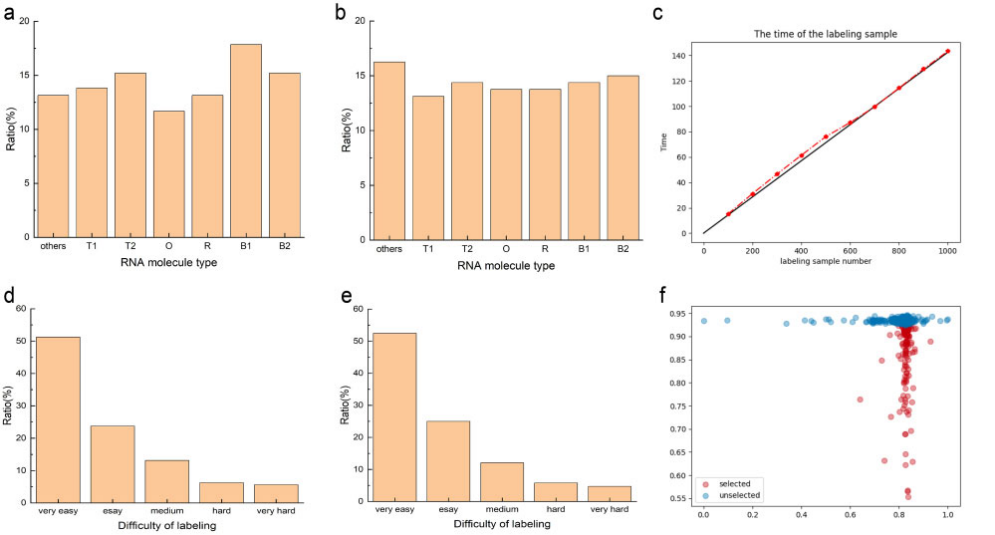
The experimental results of assessing labeling cost. (**a**) The label information distribution of the whole dataset. (**b**) The label information distribution of the selected sample by the AL strategy. (**c**) The curve of labeling time with the increase of sample size, the unit of time is minutes. (**d**) Five levels of label complexity distribution of the whole dataset. (**e**) Five levels of label complexity distribution of the selected sample by the AL strategy. (**f**) The feature value distribution of the selected sample and the unselected sample

To evaluate the effectiveness of the AL strategy on labeling costs, we experiment on RNA-CD with the UNC AL strategy. The biologists label the entire RNA-CD label and selected samples using the AL strategy. At the same time, they evaluate the complexity of labeling. In the experimental configuration, we set five difficulty indicators: ‘Very easy’, ‘Easy’, ‘Medium’, ‘Hard’ and ‘Very hard’. The RNA-CD labeling information is shown in Figure 6a, and the complexity of the whole RNA-CD is shown in Figure 6d. The total labeling time for RNA-CD is 2h27min09s with a dataset of 1020 samples. The curve of labeling time is shown in Figure 6c. In the beginning, the labeling samples need more time to explore the labeling details. When using the AL strategy, 160 of the 15.67% of the whole data volume is selected.

We use two sets of experimental data, namely the professional labeling of experts in the nanopore field and labeling of non-experts by simple learning. The labeling results show that experts in the nanopore field label relatively quickly, while non-experts take longer to label. For samples with different difficulties in the sample labeling process, the labeling experts perform subjective analysis according to the previously labeled sample rules. Inaccurate labeling occurs in the labeling of samples or the labelers get tired during labeling process and the samples are labeled incorrectly. Therefore, a small number of samples will be mislabeled in all labeled samples.

Similarly, biologists need to label the selected samples again and evaluate the complexity of the selected samples. The labeling information of the selected samples is shown in Figure 6b. To acknowledge the difficulty level of the selected samples, the complexity of the selected samples is shown in Figure 6e. The AL strategy selects some relatively complex samples that can be easily mislabeled, which can improve the generalization ability of the model for these unseen samples in the test dataset. The labeling time of the AL strategy is about 17min56s, which is about 12.24% of the whole data labeling time. It should be noted that the AL strategy can save more sample labeling cost. The selected samples have the same feature value as shown in Figure 6f, but the features of the unselected samples are different. It is proved that the AL strategy can select the samples with the same feature value.

## 4 Conclusion

In this work, we apply active learning to the nanopore field. We verify the active learning strategies on the RNA type classification and ONT barcode datasets. The experimental results show that the active learning strategies can drastically reduce the labeling cost. Moreover, in the machine learning phase, the active learning strategies can help nanopore specialists understand which samples are crucial for the classification task. The extent to which active learning can be applied to nanopore dataset analysis has yet to be fully demonstrated, but we believe that the initial validation performed in this work is promising for future applications. This work intends to inform biologists on how to understand and utilize active learning technology.

## Supplementary Material

btac764_Supplementary_Data

## Data Availability

The datasets generated during and/or analyzed during the current study are available from the corresponding author on reasonable request.

## References

[btac764-B1] Aksimentiev A. et al (2004) Microscopic kinetics of DNA translocation through synthetic nanopores. Biophys. J., 87, 2086–2097. 15345583 10.1529/biophysj.104.042960 PMC1304610

[btac764-B2] Balcan M.-F. et al (2009) Agnostic active learning. J. Comput. Syst. Sci., 75, 78–89.

[btac764-B3] Bell N.A. , Keyser U.F. (2016) Digitally encoded DNA nanostructures for multiplexed, single-molecule protein sensing with nanopores. Nat. Nanotechnol., 11, 645–651. 27043197 10.1038/nnano.2016.50

[btac764-B4] Beluch W.H. et al (2018) The power of ensembles for active learning in image classification. In: *Proceedings of the IEEE Conference on Computer Vision and Pattern Recognition, Salt Lake City, USA*. IEEE, pp. 9368–9377.

[btac764-B5] Castro-Wallace S.L. et al (2017) Nanopore DNA sequencing and genome assembly on the international space station. Sci. Rep., 7, 1–12. 29269933 10.1038/s41598-017-18364-0 PMC5740133

[btac764-B6] Collins B. et al (2008) Towards scalable dataset construction: an active learning approach. In: *European Conference on Computer Vision, Marseille, France*. Springer, pp. 86–98.

[btac764-B7] Duplyakin D. et al (2016) Active learning in performance analysis. In: *2016 IEEE International Conference on Cluster Computing (CLUSTER), Taipei, China*. IEEE, pp. 182–191.

[btac764-B8] Farquhar S. et al (2021) On statistical bias in active learning: how and when to fix it. In: *International Conference on Learning Representations, Online.*

[btac764-B9] Farshad M. , Rasaiah J.C. (2020) Molecular dynamics simulation study of transverse and longitudinal ionic currents in solid-state nanopore DNA sequencing. ACS Appl. Nano Mater., 3, 1438–1447.

[btac764-B10] Feng Y. et al (2015) Nanopore-based fourth-generation DNA sequencing technology. Genomics, Proteomics Bioinformatics, 13, 4–16. 25743089 10.1016/j.gpb.2015.01.009 PMC4411503

[btac764-B11] Freund Y. et al (1997) Selective sampling using the query by committee algorithm. Mach. Learn., 28, 133–168.

[btac764-B12] Gal Y. et al (2017) Deep Bayesian active learning with image data. In: *International Conference on Machine Learning, Sydney, Australia*. PMLR, pp. 1183–1192.

[btac764-B13] Gong Y. et al (2021) DeepReac+: deep active learning for quantitative modeling of organic chemical reactions. Chem. Sci., 12, 14459–14472. 34880997 10.1039/d1sc02087k PMC8580052

[btac764-B14] Guan X. et al (2022) S2Snet: deep learning for low molecular weight RNA identification with nanopore. Brief. Bioinformatics, 23(3), bbac098. 10.1093/bib/bbac098 35368061

[btac764-B15] Henley R.Y. et al (2016) Electrophoretic deformation of individual transfer RNA molecules reveals their identity. Nano Lett., 16, 138–144. 26609994 10.1021/acs.nanolett.5b03331 PMC4890568

[btac764-B17] Hoenen T. et al (2016) Nanopore sequencing as a rapidly deployable ebola outbreak tool. Emerg. Infect. Dis., 22, 331–334. 26812583 10.3201/eid2202.151796 PMC4734547

[btac764-B18] Huang S.-J. et al (2010) Active learning by querying informative and representative examples. In: *Advances in Neural Information Processing Systems, Vancouver, B.C., Canada*, Vol. 23. pp. 892–900.

[btac764-B19] Jablonka K.M. et al (2021) Bias free multiobjective active learning for materials design and discovery. Nat. Commun., 12, 1–10. 33875649 10.1038/s41467-021-22437-0 PMC8055971

[btac764-B20] Jia S. et al (2019) Detection of m 6 A RNA methylation in nanopore sequencing data using support vector machine. In: *2019 12th International Congress on Image and Signal Processing, BioMedical Engineering and Informatics (CISP-BMEI), Suzhou, China*. IEEE, pp. 1–5.

[btac764-B21] Johnson S.S. et al (2017) Real-time DNA sequencing in the antarctic dry valleys using the oxford nanopore sequencer. J. Biomol. Tech., 28, 2–7. 28337073 10.7171/jbt.17-2801-009 PMC5362188

[btac764-B22] Joshi A.J. et al (2009) Multi-class active learning for image classification. In: *2009 IEEE Conference on Computer Vision and Pattern Recognition, Miami, Florida, USA*. IEEE, pp. 2372–2379.

[btac764-B23] Kasianowicz J.J. et al (1996) Characterization of individual polynucleotide molecules using a membrane channel. Proc. Natl. Acad. Sci. USA, 93, 13770–13773. 8943010 10.1073/pnas.93.24.13770 PMC19421

[btac764-B26] Kolmogorov M. et al (2017) Single-molecule protein identification by Sub-nanopore sensors. PLoS Comput. Biol., 13, e1005356. 28486472 10.1371/journal.pcbi.1005356 PMC5423552

[btac764-B27] Konyushkova K. et al (2017) Learning active learning from data. In: *Advances in Neural Information Processing Systems, Long Beach, California, USA*, Vol. 30.

[btac764-B28] Kusne A.G. et al (2020) On-the-fly closed-loop materials discovery via Bayesian active learning. Nat. Commun., 11, 1–11. 33235197 10.1038/s41467-020-19597-w PMC7686338

[btac764-B29] Laver T. et al (2015) Assessing the performance of the oxford nanopore technologies minion. Biomol. Detect. Quant., 3, 1–8. 10.1016/j.bdq.2015.02.001 PMC4691839 26753127

[btac764-B30] Lewis,D.D. (1995) A sequential algorithm for training text classifiers: Corrigendum and additional data. In: *ACM SIGIR Forum, New York, USA*, Vol. 29. pp. 13–19.

[btac764-B31] Liu H. et al (2019) Accurate detection of m 6 a RNA modifications in native RNA sequences. Nat. Commun., 10, 1–9. 31501426 10.1038/s41467-019-11713-9 PMC6734003

[btac764-B32] Liu Q. et al (2019) Detection of DNA base modifications by deep recurrent neural network on oxford nanopore sequencing data. Nat. Commun., 10, 2449. 31164644 10.1038/s41467-019-10168-2 PMC6547721

[btac764-B33] Lookman T. et al (2019) Active learning in materials science with emphasis on adaptive sampling using uncertainties for targeted design. NPJ Comput. Mater., 5, 1–17.

[btac764-B34] Loose M. et al (2016) Real-time selective sequencing using nanopore technology. Nat. Methods, 13, 751–754. 27454285 10.1038/nmeth.3930 PMC5008457

[btac764-B35] Mahapatra D. et al (2018) Efficient active learning for image classification and segmentation using a sample selection and conditional generative adversarial network. In: *International Conference on Medical Image Computing and Computer-Assisted Intervention, Granada, Spain*. Springer, pp. 580–588.

[btac764-B36] Majd S. et al (2010) Applications of biological pores in nanomedicine, sensing, and nanoelectronics. Curr. Opin. Biotechnol., 21, 439–476. 20561776 10.1016/j.copbio.2010.05.002 PMC3121537

[btac764-B37] Mayer C. , Timofte R. (2020) Adversarial sampling for active learning. In: *Proceedings of the IEEE/CVF Winter Conference on Applications of Computer Vision, Snowmass village, Colorado, USA*. pp. 3071–3079.

[btac764-B38] Misiunas K. et al (2018) QuipuNet: convolutional neural network for single-molecule nanopore sensing. Nano Lett., 18, 4040–4045. 29845855 10.1021/acs.nanolett.8b01709 PMC6025884

[btac764-B39] Nguyen H.T. , Smeulders A. (2004) Active learning using pre-clustering. In: *Proceedings of the Twenty-first International Conference on Machine Learning, Banff, Alberta, Canada.* pp. 79.

[btac764-B40] Ni P. et al (2019) DeepSignal: detecting DNA methylation state from nanopore sequencing reads using deep-learning. Bioinformatics, 35, 4586-4595. 10.1093/bioinformatics/btz276 30994904

[btac764-B41] Roy N. , McCallum A. (2001) Toward optimal active learning through Monte Carlo estimation of error reduction. In: *ICML, Williams College, Williamstown, MA, USA*, Vol. 2. pp. 441–448.

[btac764-B42] Schreiber J. , Karplus K. (2015) Analysis of nanopore data using hidden markov models. Bioinformatics, 31, 1897–1903. 25649617 10.1093/bioinformatics/btv046 PMC4553831

[btac764-B43] Sener O. , Savarese S. (2018) Active learning for convolutional neural networks: a core-set approach. S*tat.*, 1050, 21.

[btac764-B44] Sinha S. et al (2019) Variational adversarial active learning. In: *Proceedings of the IEEE/CVF International Conference on Computer Vision, Seoul, South Korea*. IEEE. pp. 5972–5981.

[btac764-B45] Smith A.M. et al (2015) Capture, unfolding, and detection of individual tRNA molecules using a nanopore device. Front. Bioeng. Biotechnol., 3, 91. 26157798 10.3389/fbioe.2015.00091 PMC4478443

[btac764-B46] Smith J.S. et al (2018) Less is more: sampling chemical space with active learning. J. Chem. Phys., 148, 241733. 29960353 10.1063/1.5023802

[btac764-B47] Smith, M. A. et al. (2020) Molecular barcoding of native RNAs using nanopore sequencing and deep learning. *Genome Res.*, **30**, 1345–1353. 10.1101/gr.260836.120 PMC7545146 32907883

[btac764-B48] Steinbock L. et al (2014) Probing the size of proteins with glass nanopores. Nanoscale, 6, 14380–14387. 25329813 10.1039/c4nr05001k

[btac764-B49] Tang Y.-P. , Huang S.-J. (2019) Self-paced active learning: query the right thing at the right time. In: *Proceedings of the AAAI Conference on Artificial Intelligence, Hawaii, USA*, Vol. 33. pp. 5117–5124.

[btac764-B50] Tang Y.-P. et al (2019) ALiPy: active learning in python. *arXiv preprint arXiv:1901.03802*.

[btac764-B51] Traversi F. et al (2013) Detecting the translocation of DNA through a nanopore using graphene nanoribbons. Nat. Nanotechnol., 8, 939–945. 24240429 10.1038/nnano.2013.240

[btac764-B52] Ueno T. et al (2021) Automated stopping criterion for spectral measurements with active learning. NPJ Comput. Mater., 7, 1–9.

[btac764-B53] Wang K. et al (2017) Cost-effective active learning for deep image classification. IEEE Trans. Circuits Syst. Video Technol., 27, 2591–2600.

[btac764-B54] Wang Y. et al (2021) Structural-profiling of low molecular weight RNAs by nanopore trapping/translocation using *Mycobacterium smegmatis* porin A. Nat. Commun., 12, 3368. 34099723 10.1038/s41467-021-23764-y PMC8185011

[btac764-B55] Wang Y. et al (2019) Nanopore sequencing accurately identifies the mutagenic DNA lesion O6‐carboxymethyl guanine and reveals its behavior in replication. Angew. Chem., 131, 8520–8524. 10.1002/anie.201902521 31021463

[btac764-B56] Xin R. et al (2021) Active-learning-based generative design for the discovery of wide-band-gap materials. J. Phys. Chem. C, 125, 16118–16128.

[btac764-B57] Ying Y.L. et al (2014) Single molecule analysis by biological nanopore sensors. Analyst, 139, 3826–3835. 24991734 10.1039/c4an00706a

[btac764-B58] Yoo D. , Kweon I.S. (2019) Learning loss for active learning. In: *Proceedings of the IEEE/CVF Conference on Computer Vision and Pattern Recognition, Long Beach, CA, USA*. pp. 93–102.

[btac764-B59] Zhang B. et al (2020) State-relabeling adversarial active learning. In: *Proceedings of the IEEE/CVF Conference on Computer Vision and Pattern Recognition, Seattle, WA, USA*. IEEE. pp. 8756–8765.

[btac764-B60] Zhang X. et al (2015) Mimicking ribosomal unfolding of RNA pseudoknot in a protein channel. J. Am. Chem. Soc., 137, 15742–15752. 26595106 10.1021/jacs.5b07910 PMC4886178

[btac764-B61] Zhang X. et al (2017) Nanopore electric snapshots of an RNA tertiary folding pathway. Nat. Commun., 8, 1–11. 29133841 10.1038/s41467-017-01588-z PMC5684407

