# Supplementary material for: Active learning for efficient analysis of high-throughput nanopore data
Source: Bioinformatics. 2022 Nov 29;39(1):btac764. doi: 10.1093/bioinformatics/btac764 (PMC9825740; doi:10.1093/bioinformatics/btac764)
Supplement: btac764_Supplementary_Data [file btac764_supplementary_data.zip › SI-active _learning1 .docx]

## Supporting Information

## A Data Distribution

Fig.S1 shows the sample distribution from RNA-CD. Fig. S1a to Fig. S1g show the double-histogram of the seven RNA sequencing signals. The horizontal axis is the sequence length and the vertical axis is the ratio of the open-pore current. Fig. S1a shows the distribution of the noise signals, which are widely distributed in the number range from 0 to 0.5, and a few signals are at minus 0.5. Obviously, the noise signal is a special type of signal without any pattern. The sequencing signals show that tRNA type 1, overhanged siRNA, blunt siRNA type 1, and blunt siRNA type 2 have the same signal shape, but the amplitude values are different. Specifically, Fig. S1b, Fig. S1d, Fig. S1f, and Fig. S1g show the distribution of the above four RNA signals, which are widely distributed at 0.45, 0.42, 0.48, and 0.5, respectively. In contrast, Fig. S1c shows the distribution of the tRNA type 2 signal, which is widely distributed between 0.55 and 0. Fig. S1e shows the distribution of the 5S rRNA, which is widely distributed in the range of values between 0 and 0.65.

To obtain the ideal result for the classification task, we extracted eleven features of each sequence from the statistical information, including the rate of change of amplitude (noise), dwell time (length), mean, standard deviation (std), kurtosis (kurt) and skewness (skew). Fig. S1h shows the scatter plot of the feature “noise” and the feature “length”, and Fig. S1i shows the scatter plot of the feature “std” and the feature “length”. The red dots are marked as noise and the orange dots are marked as the other six RNA categories. It is obvious that any two features are insufficient to satisfy the final classification conditions. Therefore, all eleven features must be used together to obtain the ideal result.


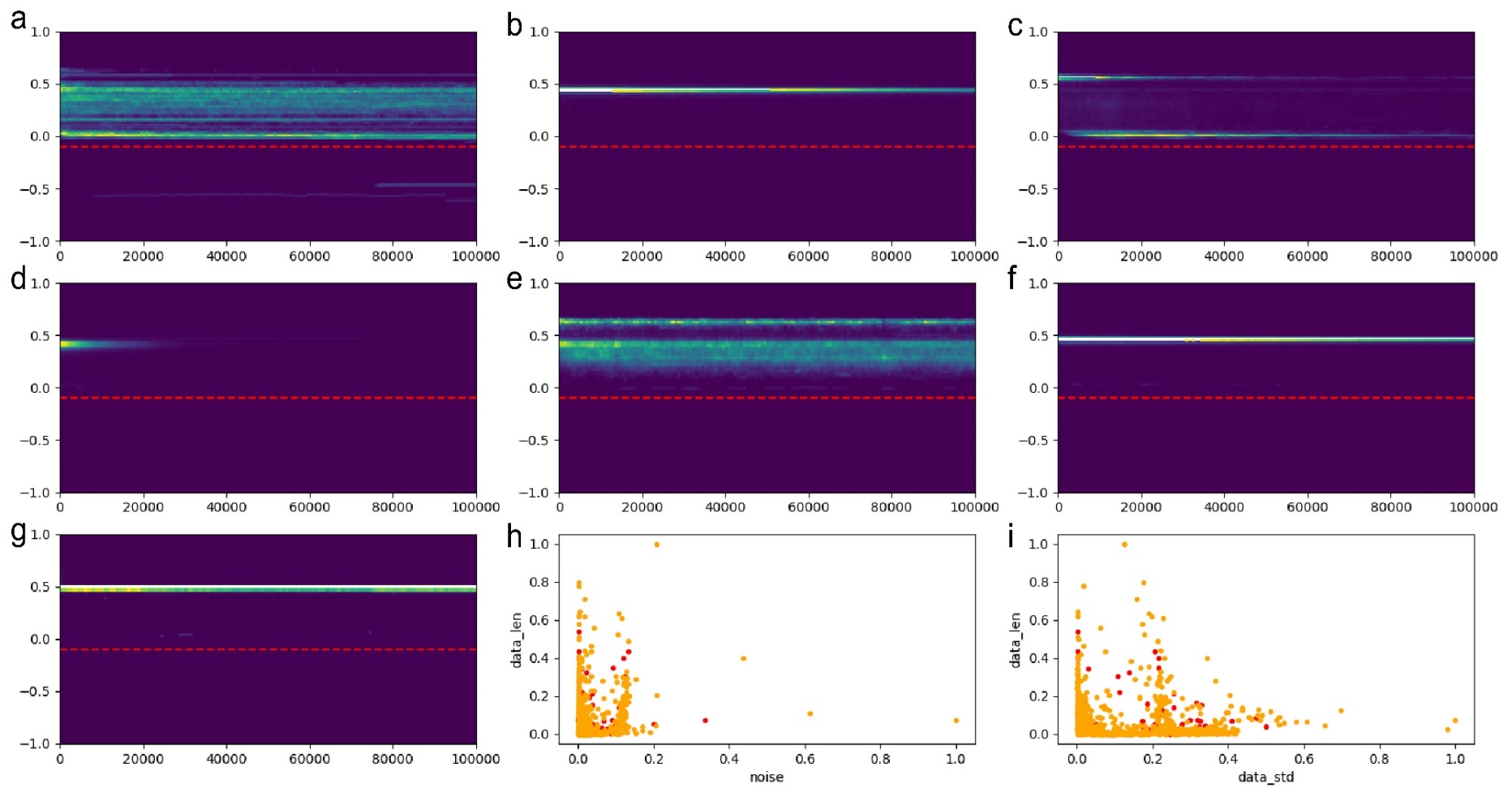


**Fig. S1.** **The visualization of sample distribution in RNA-CD. a** to **g** are the RNA raw sample distribution of noise, tRNA type 1, tRNA type 2, overhanged siRNA, 5S rRNA, blunt siRNA type 1, and blunt siRNA type 2, respectively. **h** and **i** are the scatter plots between the two features.

## B Feature Distribution

The features we extract are 11-dimensional feature vectors that must be normalized by the Min-Max Scaling to map data between 0 and 1. We use the “*distplot-histogram*” to obtain the distribution of the normalized data, as shown in Fig.S2. Fig.S2a, Fig.S2c, and Fig.S2f show that the feature distribution of noise, standard deviation, and length, which have a wide range. These distributions are comparative scatter, which can be considered as a crucial feature for classification. In contrast, Fig.S2b, Fig.S2d, Fig.S2e, Fig.S2g, Fig.S2h, and Fig.S2i show the feature distribution of skewness, maximum, minimum, kurtosis, level 2 position, and level 1 position, which have a small range. The more concentrated the sample, the narrower the sample distribution, making the corresponding features more difficult to classify.

This can be considered as a guide for selecting the sample to select the more discriminative and representative features considering the outer distribution of the features in the training dataset – so the model learns the different samples for the test performance more effectively.
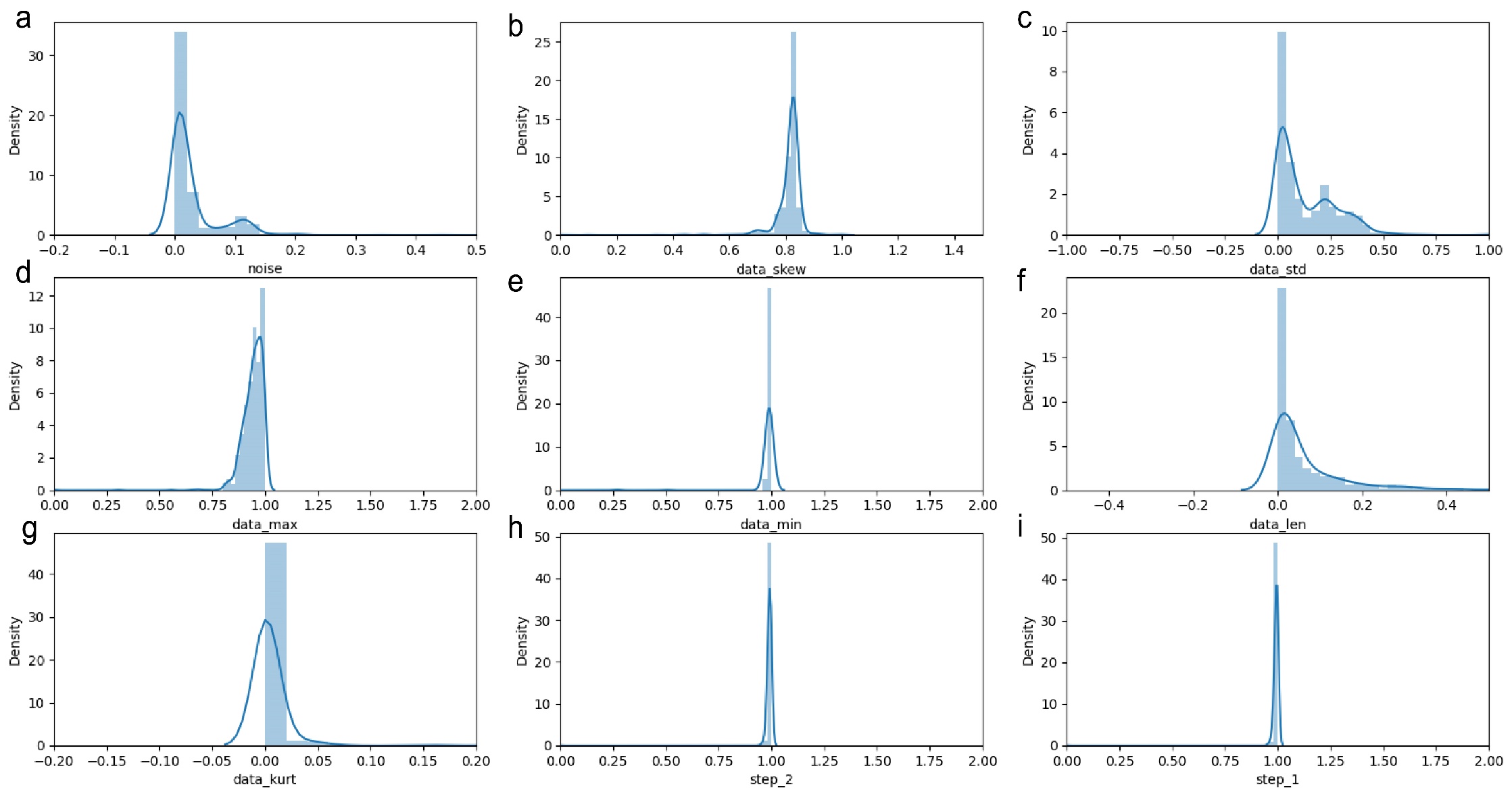


**Fig. S2.** **The visualization of features distribution in RNA data.** **Fig.S2a** to **Fig.S2i** are the feature distribution of feature noise, skewness, standard deviation, maximum, minimum, length, kurtosis, level 2 position, and level 1 position, respectively.

**C PCA scatter plot of the RNA dataset**

Fig.S3 shows the PCA distribution of the RNA datasets. From the figure, it can be seen that a simple PCA mapping is performed, but it is still difficult to make a simple classification of the samples based on the classification boundary. Especially in the noise class, there are many outliers, which lead to many errors in classification.


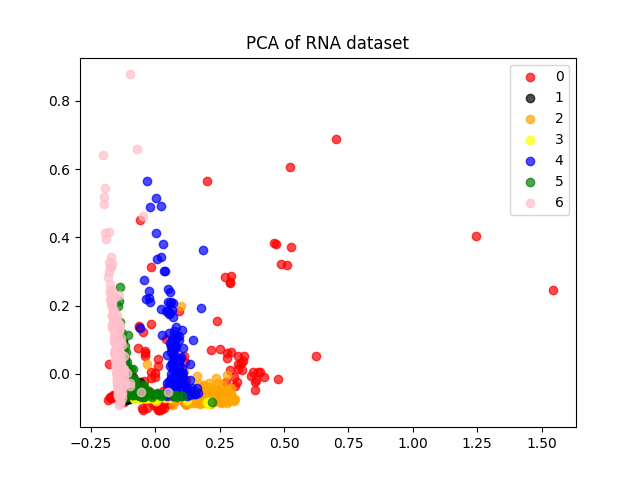


**Fig. S3. The PCA scatter plot of the RNA dataset**. Categories 0 through 6 are noise, tRNA type 1, tRNA type 2, overhanged siRNA, 5S rRNA, blunt siRNA type 1, and blunt siRNA type 2, respectively.

**D The compared of features distribution in RNA data**

Fig. S4 shows the feature distributions for the RNA dataset before and after sample selection. From Fig.S4a to Fig.S4i, it can be seen that the 11 features used in the previous work all follow one criterion, i.e., the selected and unselected samples all have the same distribution. This also shows that active learning can effectively select samples that can be better learned by classifiers, thus achieving the purpose of reducing the cost of sample labeling.


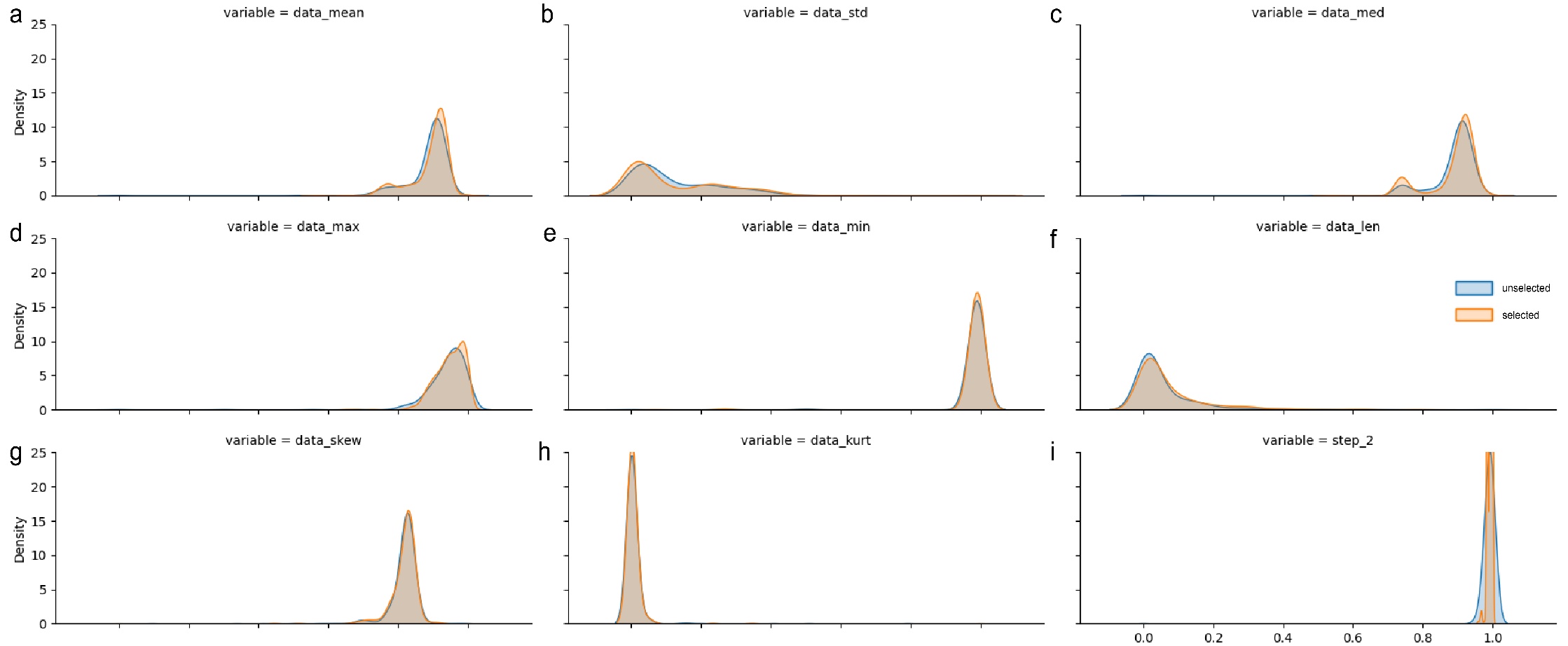


**Fig. S4. The compared of features distribution between the unselected samples and selected samples in RNA data.** **Fig.S4a** to **Fig.S4i** are the feature distribution of feature mean, standard deviation, medium, maximum, minimum, length, skewness, kurtosis, and level 2 position, respectively.

**E Confusion Matrix**

Fig.S5a and Fig.S5b show the confusion matrix results for the performance of the sample training model selected by UNC for RNA-CD. The results of the RF method are shown in Fig. S5a. It can be seen that the accuracy of overhanged siRNA, blunt siRNA type 1 and siRNA type 2, tRNA type 1 and type 2, 5S rRNA are 0.9694, 0.9630, 0.9206, 0.9600, 0.9079 and 0.9118 respectively. Compared to our previous work ([Wang, et al., 2021](#_ENREF_3)), there is a significant improvement in the “Other” category, showing that UNC strategy selects samples that are more likely to be misclassified (“Other” category), to achieve the goal of training a small number of samples to achieve optimal performance. The results of the S2Snet method are shown in Fig. S5b. The accuracy of overhanged siRNA, blunt siRNA type 1 and siRNA type 2, tRNA type 1 and type 2, 5S rRNA are 0.9845, 0.9865, 0.9802, 0.9832, 0.9654 and 0.9476 respectively. Similar to the results of RF (Fig. S5a), the “other” category is significantly improved compared with our previous work ([Guan, et al., 2022](#_ENREF_1)). Fig. S5c shows the confusion matrix of the QuipuNet model for the test set after training when the samples selected by UNC active learning for the ONT-BD dataset achieve the optimal performance. As can be seen from the figure, compared to the previous work ([Misiunas, et al., 2018](#_ENREF_2)), the samples selected by UNC improved the accuracy of “100” and “110”, which are easily misclassified. The results are similar to the previous RNA-CD datasets.


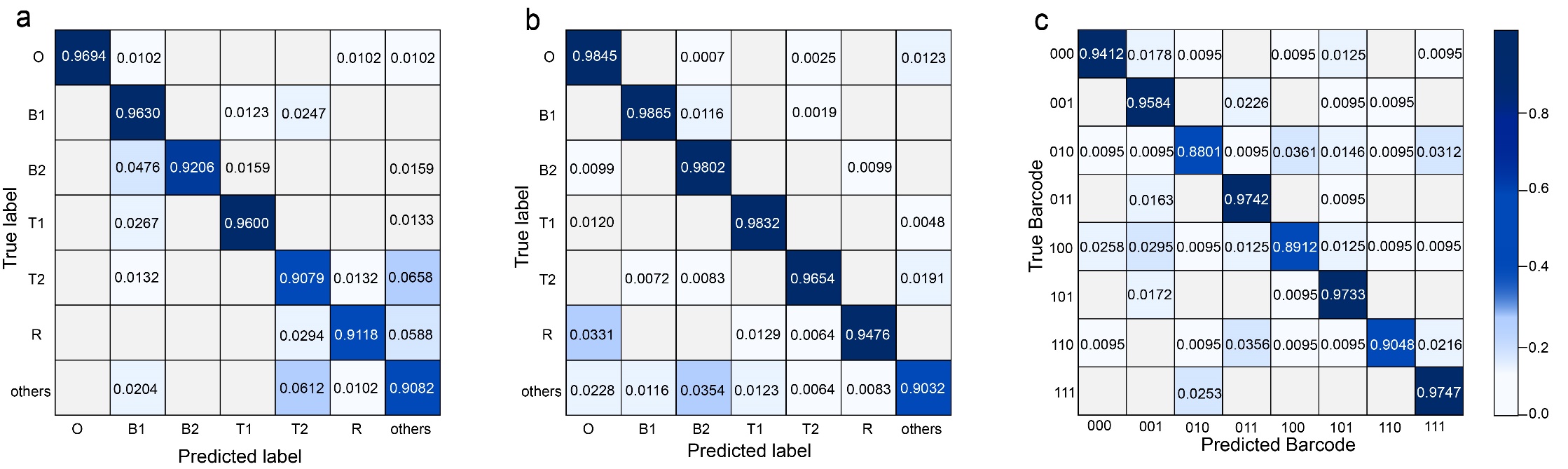


**Fig. S5. The results of the confusion matrix.** **a.** the results of the confusion matrix of the RF model trained with the selected sample by UNC which achieves the performance of the full ground truth training at RNA-CD. **b.** the results of confusion matrix of the S2Snet model trained with the selected sample model by UNC which achieves the performance of the full ground truth training at RNA-CD. The RNA types are overhanged siRNA (O), blunt siRNA type 1 (B1), blunt siRNA type 2 (B2), tRNA type 1 (T1), tRNA type 2 (T2), 5S rRNA (R). **c.** the results of confusion matrix of the QuipuNet model trained with selected sample by UNC which achieves the performance of the full ground truth training at ONT-BD.

**F Pseudo Code of Computing the Bias Constraint**

Due to the particularity of nanopore data, some samples are difficult to label. The direct application of active learning algorithm affects the performance of the model. In order to further improve the accuracy of the model, we propose a concept called bias constraint, which further optimizes the process of active learning algorithm selecting samples by assigning weights to unlabeled samples. Here we briefly describe the calculation process of bias constraint. We show the pseudocode in Algorithm 1.

| **Algorithm 1:** Compute the Bias Constraint |
| --- |
| **Input :** The initial training dataset: $D_{train}=\left\{ t_{m} \vert1\leq m\leq M \right\}$; The unlabeled dataset: $D_{pool}=\left\{ x_{n} \vert1\leq n\leq N \right\}$; The test dataset $D_{test}$:The number of samples selected per iteration: Z.  **Intermediate:** Center point after t-SNE feature mapping of training dataset: O; Samples selected by active learning: $S=\left\{ s_{z} \vert1\leq z\leq Z \right\}$ |
| **1** **Begin**  **2** Model←training($D_{train}$)  **3** **While** Model($D_{test}$) is not the best performance:  **4** **\|** O←Center(t-SNE($D_{train}$));  **5** **\|** **for** i = 1, 2, …, N do  **6** **\|** **\|** $w_{i}$ ← Distance(t-SNE($x_{i}$),O);  **7** **\|** \| *W*← Append(*W*, $w_{i}$);  **8 \| end**  **9 \|** *W*←1-Normalization(*W*)  **10** **\|** $S$ ← Active Learning($\mathbf{D}_{\boldsymbol{pool}}^{\boldsymbol{'}}=\left\{ {(x}_{n},w_{n}) \vert1\leq n\leq N \right\}$);  11 **\|** $D_{train}$←Append($D_{train}$,$S$);  **12 \|** Model←training($D_{train}$);  **13** **end**  14 **End** |

References

Guan, X.*, et al.* S2Snet: deep learning for low molecular weight RNA identification with nanopore. *Briefings in Bioinformatics* 2022;23(3):bbac098.

Misiunas, K., Ermann, N. and Keyser, U.F. QuipuNet: convolutional neural network for single-molecule nanopore sensing. *Nano letters* 2018;18(6):4040-4045.

Wang, Y.*, et al.* Structural-profiling of low molecular weight RNAs by nanopore trapping/translocation using Mycobacterium smegmatis porin A. *Nature Communications* 2021;12(1):3368.
