# Supplementary figures and images for: Active learning for efficient analysis of high-throughput nanopore data
Source: Bioinformatics. 2022 Nov 29;39(1):btac764. doi: 10.1093/bioinformatics/btac764 (PMC9825740; doi:10.1093/bioinformatics/btac764)

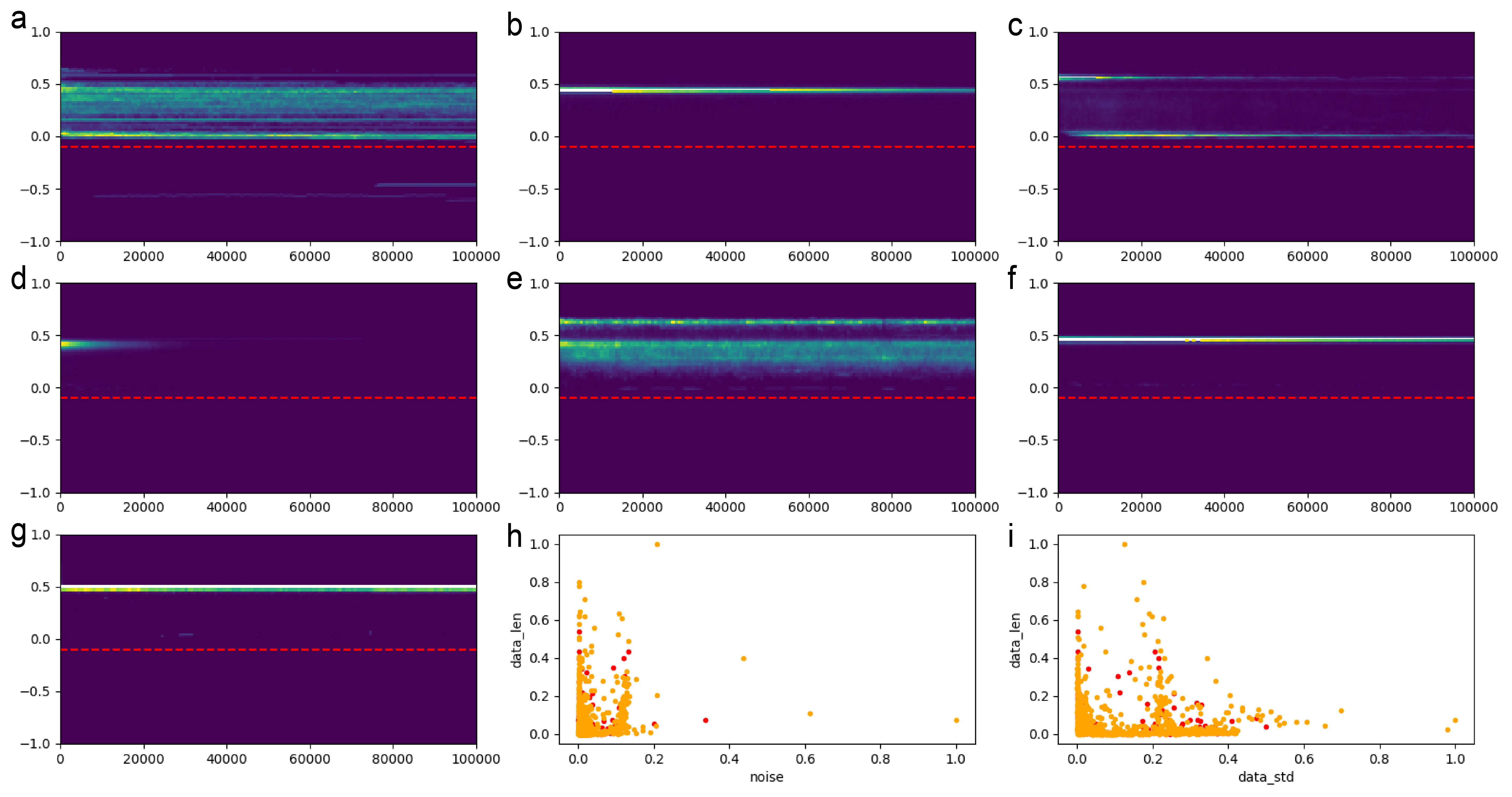

Supplement: btac764_Supplementary_Data [file btac764_supplementary_data.zip › figs1.jpg]

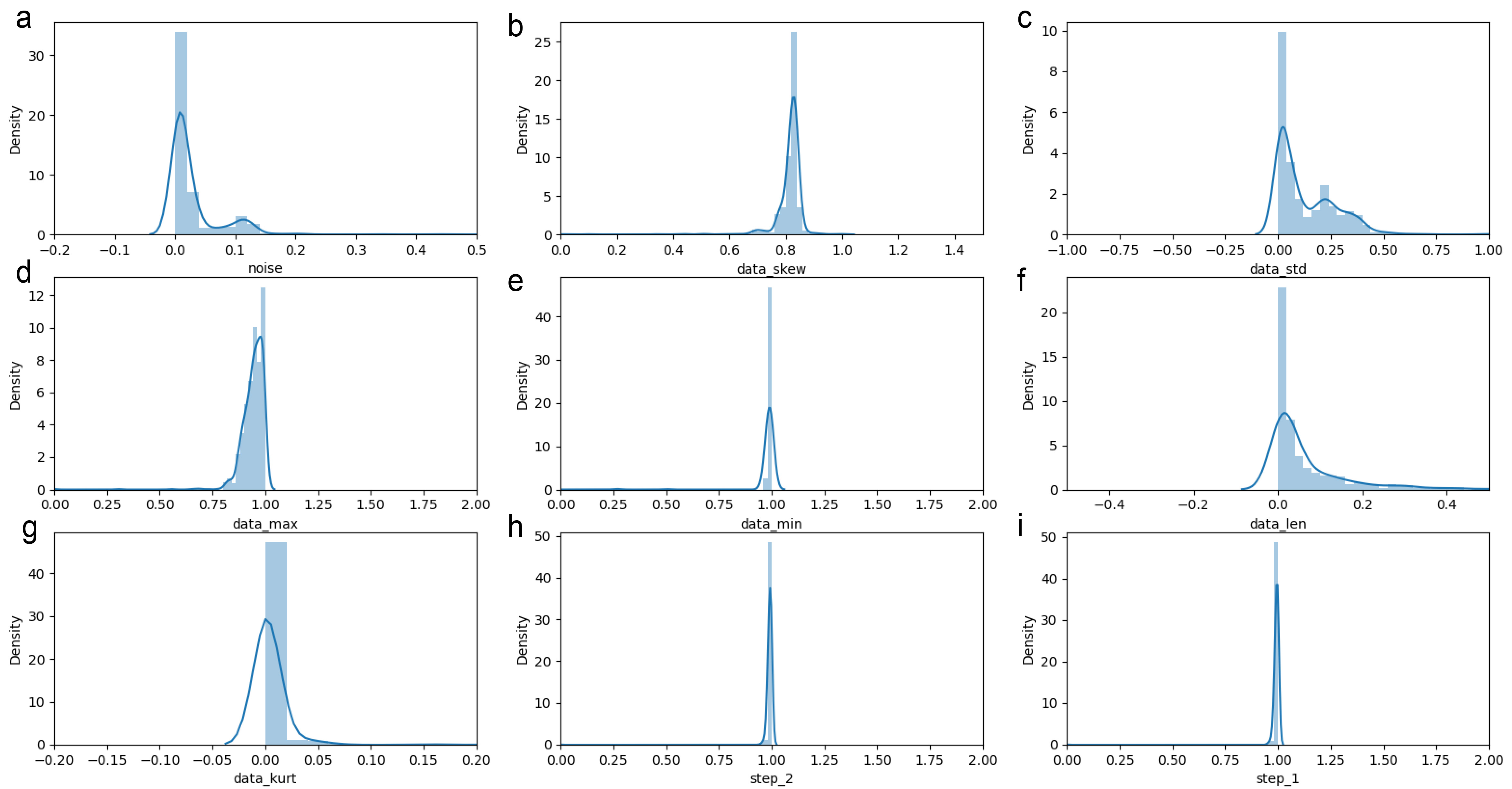

Supplement: btac764_Supplementary_Data [file btac764_supplementary_data.zip › figs2.jpg]

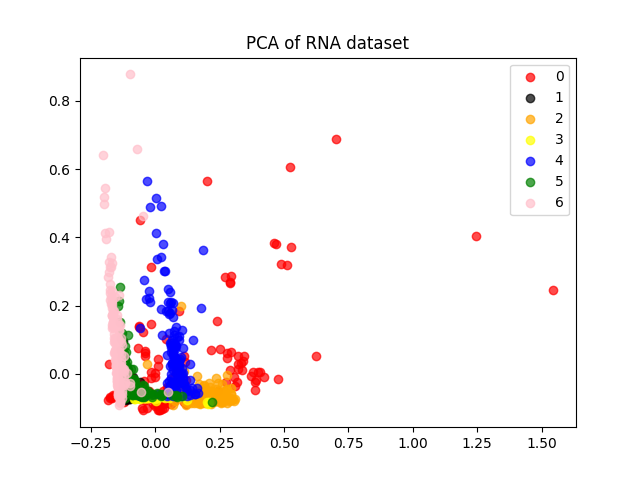

Supplement: btac764_Supplementary_Data [file btac764_supplementary_data.zip › figs3.png]

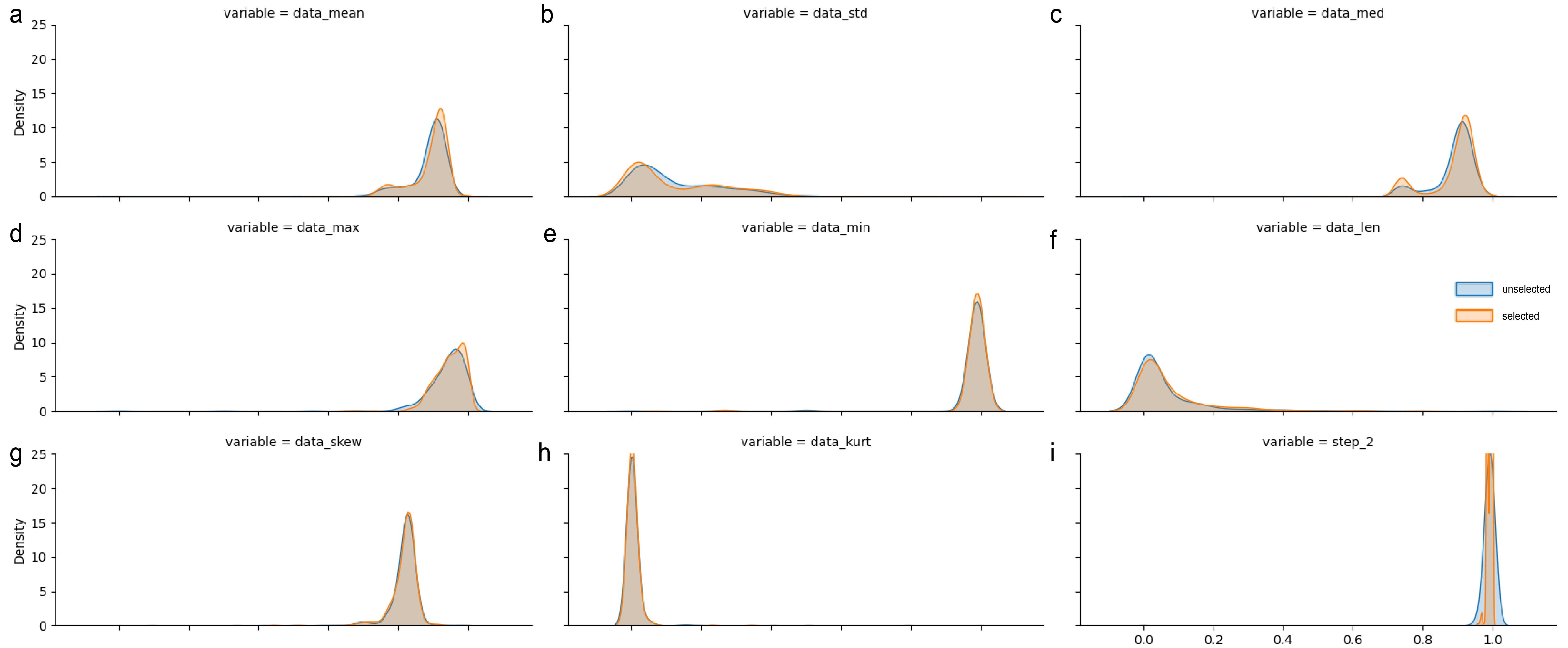

Supplement: btac764_Supplementary_Data [file btac764_supplementary_data.zip › figs4.jpg]

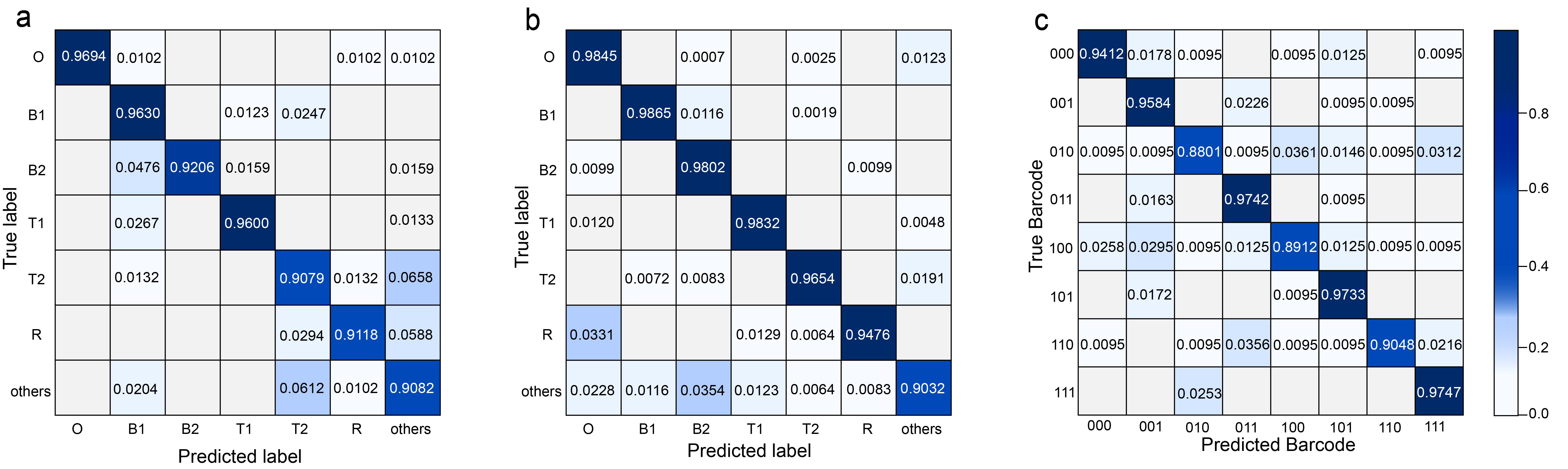

Supplement: btac764_Supplementary_Data [file btac764_supplementary_data.zip › figs5.jpg]
